# Borden’s Condensed Milk

**Published:** 1857-12

**Authors:** 


We copy from the American Medical Gazette, the portion of the report of the Section on Materia Medica and Botany, to whom was referred the subject of condensed milk. The first part of the report has reference to the constitution and physiology of milk, and we have retained only the portion which refers to the condensed milk. F.

"It affords the Section no small degree of satisfaction to be able to state to the academy that, after a thorough examination of this subject, they are fully convinced that in 'Borden's Condensed Milk' the citizens of New York may be furnished with an article that for purity, durability, and economy, is hitherto unequaled in the annals of the milk trade. The facilities that he enjoys, both in a pecuniary and scientific point of view, added to the high character he sustains as an honest, upright man, not only afford him no inducement for deception, but on the contrary, should be sufficient to inspire all with implicit confidence in the truth of his statements, and insure the final success of his undertaking.

"In order to carry out his views to the greatest advantage both to himself and patrons, he has selected for the seat of his operations, a section of country abounding in rich pastures and healthy cattle, and so remote from the city as to enable him to procure milk from the neighboring farmers at a much cheaper rate than is usually paid by the milk dealers. As soon as received, it is with as little delay as possible deprived of a greater part of its watery portion, leaving the nutritious portion wholly unaffected, and in a portable condition. In the examination of the merits of this article, the section have thought proper to acquaint themselves, as far as possible, with the mode of its preparation, to test its value as an article of diet, its therapeutic virtues, its purity, its durability, and economy.

"In order to become more thoroughly satisfied concerning the manner in which this milk is prepared, it was thought advisable that the whole process be submitted to the careful inspection of some member of the section. Accordingly, the secretary, accompanied by Dr. Griscom, (who has kindly volunteered his assistance in the investigation of this most interesting subject,) at their earliest convenience, visited the laboratory of Messrs. Borden & Co., and were enabled to witness the process through its different stages, from the milking of the cows to its final completion. The milk, immediately after leaving the cow, was strained into an ordinary milk can, then placed in a cold water bath, and there it remained until it was entirely deprived of its animal heat. It was then placed in a hot bath, and soon as possible raised to a temperature of about 175° Fahrenheit. This caused a slight deposit of a viscid albuminous matter upon the sides of the can, which, if allowed to take place in the evaporating pan, greatly retards the process of evaporation. The milk is now passed through a second strainer, and without delay removed to a vacuum pan, where the water is evaporated. This pan consists of a large metallic vessel, supplied with a jacket for the reception of steam, by means of which the heat is applied. Connected with the pan, by a tube, is a barometer that indicates at a glance the extent of vacuum, and by another tube, which serves also for the escape and condensation of steam, an air pump in constant motion. A thermometer, also in direct communication with the interior of the pan, indicates the temperature, and by means of a glass light in either side of the pan we can from time to time witness the progress of the evaporation. While witnessing the process, every few minutes a note was made of the temperature, extent of vacuum, and other points of interest. For instance, at 9 o'clock there were 247 quarts in the pan, boiling at a temperature of 118° in a vacuum of 27½ inches. At forty minutes past 9, 180 quarts were added; at thirty-five minutes past ten, 240 quarts were added, making in all 667 quarts, which by thirty-five minutes past 1 o'clock was evaporated to one fourth the quantity. During this time the temperature was twice observed to be above 130°, but most of the time it was from 115° to 126°. An opportunity was also afforded to test the relative quality of milk from the respective farms. Thus, from five different farms the milk was examined by the lactometer, and showed that the morning's milk was of a richer quality than the evening's. The cows taking little or no exercise during the night, less fat is required to support the respiration, and consequently more allowed to pass into the milk. The milk from one farm, were the cows were thin in flesh and seemed destitute of fat, was superior to the milk of cows that presented to the eye a more attractive figure. It is a fact familiar to every medical man, that those mothers who are of a slender frame, small muscular development, and deficient in adipose tissue, often afford better nourishment for their offspring than many mothers of a full and plethoric habit. In the one case the fat and casein supplied by the food passes into the milk; in the other, more of the one is deposited and retained in the areolar tissue and the other contributes to the support and development of the muscular system. During the preparation of this milk, your committee beheld nothing that was not to their minds eminently satisfactory, both in the frankness with which everything pertaining to the subject was submitted to our examination, and the intelligence manifested in the replies to our many inquiries.

"In reference to the condensed milk as an article of diet, its importance is at once established by the fact that it retains all of the nutritive qualities of milk uninjured. Cheese made from it is superior to common cheese, as it contains not only the casein and butter, (the beef and fat,) but the sugar also, which, as before stated, supplies the starchy matter of vegetable food. In the ordinary method of cheese-making, the sugar is lost in the whey, thus depriving us of a very important element of nutrition. Used in its condensed form, this milk imparts a delicious flavor to coffee, fully equal to that of thick cream, and in short, as far as could be ascertained upon the most rigid inquiry, it has, whenever used in the various departments of the culinary art, given entire satisfaction. Its therapeutic value is restricted to that of pure milk and cream, and wherever these are indicated the condensed milk is equally valuable. It has been suggested as a substitute for cod liver oil, and in those cases in which the oil is not tolerated by the stomach, and cream has fulfilled the indications presented, the same amount of benefit may be derived from its employment; but as its amount of carbonaceous matter is so much less in proportion to its volume than exists in the oil, the section are not prepared to say that it can in all cases be substituted for that remedy. The opinion prevails to a considerable extent, that boiled milk has a strong tendency to constipate the bowels, and that question has been raised in reference to the condensed milk. To a note addressed to Professor Henry G. Cox, Physician to the Nursery Hospital, where the milk has been used for the last two months, that gentleman replies that it has no such tendency, He is pleased with it as an article of diet for children in the absence of pure new milk, and he adds, 'if Mr. Borden continues to furnish it unadulterated, I think it will be a great improvement to the public over the manufactured milk now so generally in use.'

"In order to test its purity, it has been submitted to careful microscopical examinations as well as different chemical tests, and found to contain nothing but pure milk. In this connection it might be well to add, that when allowed to stand for some time, small crystals are deposited upon the bottom of the can, which has given rise to the remark, that some foreign substance had been added to the milk. These crystals are nothing but the sugar of milk, and so far from being an objection, they afford proof that the milk has undergone no decomposition. They are readily dissolved upon the addition of water. Its durability depends in a great measure upon the manner in which it is kept. When exposed to the influence of hot, damp weather, it will remain sweet but little longer than ordinary milk. If kept upon ice, or in cold weather, it will remain sweet for many weeks. When hermetically sealed, it will keep for many months, or even years. In answer to enquiries on this part of the subject, the following communications were received:

Office of N. Y. and Liverpool U. S. M. Steamers, Oct. 29, 1857.

Dr. E. H. Janes, Sec'y of Med. Com.:

Dear Sir,—In answer to your inquiries relative to Borden's Condensed Milk, I have to state that it has been used on board of this line of steamers for several voyages, and ascertaining that it would keep to Europe and back, it is put on boark for the return voyage, and found good on return of ship to this port.

G. Briggs,
Sup't of Collins' Steamships.

Steamship Augusta, October 29th, 1857.

Dr. E. H. Jones, Sec'y of Med. Com.:

Dear Sir,—In answer to your inquiries in relation to Borden's Concentrated Milk, I beg leave to state that the Steamer Augusta has used it exclusively for the last two or three months on her passage to and from Savannah, Geo., with convenience to the ship and comfort of the passengers. I

also learn that the Steamer Alabama, sailing to the same port, exclusively uses the milk.

Respectfully,

DAVID SIMMONS,
Chief Steward Steamer Augusta.

A communication was also received from Hutchinson & Co., ice dealers, stating that they had furnished steamships with this milk, and in no instance had heard any complaint of its becoming sour. Full directions are given by tho proprietor on this subject, and those who follow out faithfully these directions, will find it quite as durable as is represented. Its economy is entirely in the hands of the consumer. It is sold in its condensed form at thirty-two cents per quart. Add three quarts of water, and we have four quarts of pure cows' milk. Add another quart of water, and we have five quarts of better milk than is usually supplied at our doors. Should any one desire a still cheaper article, he can learn how to obtain it, by referring to an account of a recent meeting of milkmen, given in the New York Daily Times of Monday, Nov. 2d.

In conclusion, the section beg leave to assure the academy that they believe Borden's Condensed Milk to be what it purports to be, and nothing more, viz., pure milk deprived of most of its water, and deficient in none of its nutritive elements. They believe it to be the best possible substitute for pure new milk that can be had in this or any other city. Equally adapted to the wants of all conditions of life, and often a valuable auxilliary to the physician, either in private or hospital practice. And as such, the section would earnestly recommend it to the favorable notice of the academy.

Respectfully submitted,

JOEL FOSTER, M. D., Chairman.
E. H. JANES, M. D., Secretary.

